# The Effects of Implementation Quality of a School-Based Social and Emotional Well-Being Program on Students’ Outcomes

**DOI:** 10.3390/ejihpe10020044

**Published:** 2020-05-22

**Authors:** Katherine Dowling, Margaret M. Barry

**Affiliations:** Health Promotion Research Centre, National University of Ireland Galway, University Road, H91TK33 Galway, Ireland; margaret.barry@nuigalway.ie

**Keywords:** social and emotional learning, school-based programs, implementation quality, randomized controlled trial, mental health and well-being

## Abstract

School-based social and emotional learning (SEL) programs can be effective in producing positive outcomes for students. However, when the implementation quality is poor, these programs often lose their effectiveness and fail to produce the expected positive outcomes. The current study evaluates a school-based SEL program for 15–18-year-olds in Ireland by determining the impact of implementation quality on program outcomes. The study also examines the effects on outcomes of different implementation dimensions including Dosage, Adherence, Quality of Delivery, and Participant Responsiveness. Employing a cluster randomized controlled trial design, this study collected student outcome data (*n* = 675) from 32 disadvantaged schools across three time points (pre-, post-, 12-month follow-up) and compared these data across three treatment groups (high-implementation, low-implementation, and control). Linear mixed models (LMM) were used to determine the relationships between the implementation data and student outcome data longitudinally. The findings revealed that the positive effects of the program were only observed with the high-, but not the low-implementation group (reduced suppression of emotions (*p* = 0.049); reduced avoidance coping (*p* = 0.006); increased social support coping (*p* = 0.009); reduced levels of stress (*p* = 0.035) and depressive symptoms (*p* = 0.025). The comparison of implementation dimensions revealed that only Quality of Delivery had a significant effect on all of the tested outcomes. This study highlights the importance of high-quality implementation in producing positive outcomes and supports the need to evaluate implementation using multiple dimensions.

## 1. Introduction

Elias and colleagues [1] describe social and emotional learning (SEL) as “the process of acquiring and effectively applying the knowledge, attitudes and skills necessary to understand and manage emotions, set and achieve positive goals, appreciate the perspective of others, establish and maintain positive relationships, make responsible decisions and handle interpersonal situations constructively.” SEL has often been used as an umbrella term covering a wide range of programs and approaches and defined in several ways [2,3]. Within this study, the definition of SEL is driven by CASEL’s (Collaborative for Social and Emotional Learning) competency framework [4], and the program encapsulates the five core competencies identified within this framework: self-awareness, self-management, social awareness, relationship management, and responsible decision-making.

School-based social and emotional learning (SEL) programs have gained recognition for their ability to improve young people’s mental health and well-being through the development of social and emotional skills [5,6,7,8]. However, inconsistent and variable implementation of these programs can result in diminished or null effects for participants [9,10,11,12]. While there have been a number of reviews demonstrating the relationship between implementation quality and program outcomes [5,9,13,14], evaluation studies continue to prioritize the measurement of outcomes over implementation, and very few studies observe the direct relationship between the two [5,15,16,17,18,19]. By ignoring implementation and the conditions under which a program is delivered, it is impossible to determine what led to a program’s success or, alternatively, what caused it to fail. The absence of information on implementation could be detrimental to the future success and sustainability of SEL programs. Therefore, given that there is strong evidence demonstrating the relationship between program implementation and outcome attainment, it is essential that the systematic monitoring and evaluation of implementation is embedded as a core aspect of all program evaluation studies [9,10,19].

### 1.1. Measuring Implementation Quality

Implementation quality refers to how well a program has been delivered as intended [20,21]. Many researchers recognize that implementation quality is a multidimensional construct and, therefore, should be measured as such [9,10,19,22,23]. In some studies [24,25], “fidelity” has been conceptualized as the superordinate construct used to describe the overall pattern of implementation activity. However, in other studies, like the current one, fidelity is conceptualized in procedural terms (e.g., how closely the sequence of activities align with what was intended) and is included as a subordinate indicator alongside the other dimensions, with implementation quality seen as the superordinate construct [10,19,26]. In measuring implementation quality, Dane and Schneider [15] suggested that implementation is reflective of five core dimensions: dosage (e.g., quantity of program delivered); fidelity/adherence (e.g., how many core components were delivered as prescribed); quality of delivery (e.g., how well the facilitator delivers the program); participant responsiveness (e.g., how participants respond to or are engaged with an intervention); and program differentiation (e.g., how unique the program characteristics are compared to other programs). Although it is recognized that implementation quality consists of multiple dimensions, it is clear from the literature that more attention has been given to certain dimensions (e.g., dosage and fidelity/adherence) over others [10,27,28]. For example, in Durlak and Dupre’s [10] review of programs assessing implementation quality, they reported that 63% of the studies assessed fidelity/adherence and 49% assessed dosage, while only 10 out of the 59 studies (17%) assessed a different dimension (e.g., program reach, adaptation, or quality of delivery). A similar pattern was observed in a review by Rojas-Andrade and Bahamondes [14], which included 31 school mental health programs and found that 77% reported on fidelity/adherence, 58% on dosage, 26% on quality of delivery, and 19% on participant responsiveness. Additionally, this review found that only three studies (10%) combined dimensions to produce a total implementation composite score.

While fewer studies have examined dimensions such as quality of delivery and participant responsiveness, those that did have found that they might be equally, if not more, important for achieving program outcomes compared to dimensions such as adherence and dosage that are typically represented [9,23]. For example, in Rojas-Andrade and Bahamondes’ [14] review, they found that adherence was only weakly associated with outcome variables, whereas both quality of delivery and participant receptiveness were strongly associated with outcome achievement. Additional studies have found similar results. The Steps to Respect bullying prevention program evaluation, which was conducted in 33 primary schools in California, USA, found that adherence was not significantly associated with any of the outcomes, whereas higher levels of student engagement were related to a number of positive outcomes [29]. Another study evaluated the Keepin’ it REAL drug prevention program with 25 primary schools in Ohio, USA by observing two dimensions of implementation: adherence and delivery (combined score of teacher engagement, student engagement, and quality of delivery). The findings revealed that delivery significantly influenced substance use and norms, whereas adherence significantly predicted norms but only marginally predicted substance use [22]. Furthermore, a study of the PATHS program [26] conducted in 23 primary schools in Manchester, UK evaluated implementation quality across five dimensions: dosage, fidelity/adherence, quality of delivery, participant responsiveness, and reach. The authors found that, while higher implementation for quality of delivery and participant responsiveness resulted in lower externalizing behaviors, higher levels of program reach and fidelity were not associated with any of the outcomes. Most surprising, perhaps, was that higher levels of dosage were associated with significantly lower ratings of students’ prosocial behavior and social-emotional skills [26]. In explaining this finding, the study authors suggested a number of potential reasons, including that: (i) schools with high levels of dosage may have achieved this at the expense of other critical aspects of implementation (e.g., quality); (ii) schools with lower functioning classes and higher needs were more likely to deliver PATHS more frequently; and (iii) schools that delivered more lessons of the PATHS program spent less time on other effective approaches (e.g., targeted programs). The above studies all establish the importance of including multiple dimensions of implementation measurement. In order to advance knowledge and improve practice, as well as build on the evidence for implementation science, there needs to be a greater focus on employing multiple dimensions of implementation in order to determine the role they play in outcome achievement.

Although implementation dimensions are conceptually distinct, they are interrelated in terms of delivery. For example, a school may have high dosage but also low quality of delivery, and examining the effects of these dimensions in isolation would make it impossible to determine their joint impact on outcomes. Therefore, the implementation dimensions should be seen as interrelated but conceptually distinct indicators; therefore, it is necessary to determine the combined effect of these dimensions in order to fully understand the overall implementation quality. In an effort to measure implementation quality including multiple dimensions, previous studies have adopted an approach that uses an a priori index of indicators to calculate a cumulative total implementation index score [30]. For example, the KidsMatter program evaluation in Australia combined three dimensions of implementation quality (dosage, adherence/fidelity, and quality of delivery) to create a total index score that categorized schools into high- and low-implementation groups and assessed group allocation in relation to outcomes [30]. This method allows for the assessment of total implementation quality while also taking into account data from the multiple implementation dimensions.

### 1.2. Current Study 

This study is part of a larger cluster-randomized controlled trial of the MindOut program in post-primary schools across Ireland that involved three distinct phases. Underpinned by CASEL’s competency framework [4,31], MindOut is a universal school-based program designed to be delivered by teachers through the SPHE curriculum (Social Personal and Health Education (SPHE) is a mandatory health education curriculum in Irish schools that supports the well-being and personal skill development of students) to promote the social and emotional well-being of post-primary students aged 15–18 years. This 13-session program is delivered through a structured manual with accompanying resource materials (e.g., worksheets, PowerPoint presentations, etc.) and uses interactive teaching strategies to engage students in skill-building activities. Additional information on the program and its development can be found in the relevant literature [6,32,33,34].

A c-RCT outcomes evaluation (Phase 1) of MindOut in designated disadvantaged schools has already been published [34] and revealed that the program had significantly positive intervention effects on students’ emotional skills (e.g., coping skills, emotional regulation) and mental health (stress and depression), but found no significant impact on students’ social skills, mental well-being, and academic performance. Although the original study demonstrated positive findings, it did not account for differences and variability in the implementation quality of intervention schools.

Following the c-RCT, a mixed-methods study (Phase 2) was conducted [35] in an effort to determine implementation quality and its variability across the original intervention schools. Intervention schools (*n* = 16), were assessed on their implementation quality across four dimensions individually (Dosage, Adherence, Quality of Delivery, and Participant Responsiveness) and a composite score was used to determine a total implementation quality score (high vs. low) for each school. This study found that there was variability in the total implementation quality between schools, with scores ranging from 55% to 92% (M = 79%). Based on the total implementation score, eight schools were allocated to the high-implementation group and eight schools to the low-implementation group. The study also discovered that variability occurred within schools and across different dimensions. Of the 16 schools in this study, seven consistently scored either high (*n* = 5) or low (*n* = 2) across all four implementation dimensions, while all other schools varied, scoring high in certain dimensions and low in others. These findings highlighted the need to assess not only the relationship between total implementation quality and outcomes, but also to determine how individual dimensions moderate outcomes differently. Within the second phase of the study, a number of implementation factors (e.g., teacher factors, program factors, organizational factors, etc.) were identified by teachers and students; these are discussed in the context of relevant implementation models [10,17].

Given that the results on the outcomes of this trial have already been determined [34] and the levels of implementation quality for intervention schools have been identified [35], further investigation is required to assess the relationships between these concepts. Therefore, the aim of the current study (phase 3) is to combine the data from the previous studies (phases 1 and 2) to examine how variability in implementation moderates students’ outcomes.

Specifically, this study has two main objectives:

1. To determine whether or not the level of implementation (high/low), based on the total implementation quality score, significantly impacts program outcomes at post-intervention and 12-month follow-up when compared to the control group.
2. To examine the role that each of the four implementation dimensions of (i) Dosage, (ii) Adherence, (iii) Quality of Delivery, and (iv) Participant Responsiveness play in influencing program outcomes.

## 2. Materials and Methods 

### 2.1. Design

A cluster randomized controlled trial design was utilized for this study with three treatment groups (high-implementation, low-implementation, and control). Outcome data were assessed at baseline (winter 2016), post-intervention (spring 2017) and 12-month follow-up (spring 2018) via surveys. Implementation data were collected from intervention schools during and immediately following program delivery.

### 2.2. Participants 

Schools were randomly selected throughout the Republic of Ireland based on the criteria that they were recognized as disadvantaged (DEIS) by the Department of Education and Skills. A total of 32 schools participated in the evaluation study and these schools were randomly assigned to either the intervention (*n* = 17) or control (*n* = 15) group. All teachers in the intervention group participated in a one-day interactive training workshop, during which they received all of the training materials. This study involved students who were in 4th/Transition Year (15–17 years) or 5th year (16–18 years) at baseline. (Transition Year (TY) is a one-year optional program that exists between the Junior Certificate program (3rd year; 13–15 yrs.) and the Leaving Certificate program (6th year; 16–18yrs). Transition Year is a less structured year which gives students more space to learn, mature and develop without the presence of exam pressures). A total of 675 students responded to the questionnaires during baseline assessment. Further details on the demographic profile the sample can be found in earlier publications [27,28]. Response rates decreased at post-intervention (*n* = 497) and 12-month follow-up (*n* = 435) due to students’ absenteeism on the day of data collection or students having moved to a different school since the previous data collection.

### 2.3. Ethical Standards

Written informed consent was obtained from all individual participants included in the study and passive parental consent from all student participants was also sought. All procedures performed in the study were in accordance with the ethical standards of the National University of Ireland Galway Research Ethics Committee (ref: 16-Jul-01) and with the 1964 Helsinki Declaration and its later amendments or comparable ethical standards.

### 2.4. Measures 

#### 2.4.1. Outcome Measures 

A questionnaire was used to assess students’ social and emotional skills, mental health and well-being and academic outcomes through a number of scales, which are described below. Further detailed information on these scales (e.g., psychometric properties, scoring, example items, etc.) were reported in a previous paper by the authors [34].

Social Emotional Skills

- The Rosenberg Self-esteem Scale [36] was a 10-item scale that was originally designed for use with high school students to measure self-esteem.
- The Trait Meta-Mood Scale-24 (TMMS-24) [37], an adapted version of the original TMMS [38], was used to measure people’s ability to manage and regulate their moods and emotions (subscales: attention to feelings, emotional clarity, and emotional repair).
- The Coping Strategy Indicator (CSI-15) [39], a 15-item short form of the original 33-item scale [40], which evaluates three types of coping strategies (Subscales: Avoidance, Problem Solving, and Social Support).
- The Self-Efficacy Questionnaire (SEQ-C) [41] is a 24-item scale comprised of three main subscales (subscales: academic self-efficacy, emotional self-efficacy, and social self-efficacy); however, only the latter was utilized in this study.
- The Emotional Regulation Questionnaire (ERQ) [42] is a 10-item scale that was used to assess respondents’ (i) cognitive reappraisal and (ii) expressive suppression.
- The Adolescent Interpersonal Competence Questionnaire (AICQ) [43] assesses young people’s interpersonal skills and is composed of five subscales each with eight items (Subscales: initiating relationships, providing emotional support, self-disclosure, asserting influence, and conflict resolution). Only the two latter subscales were used for the purpose of this study.
- The Making Decisions in Everyday Life Scale five-item short form [44], an adapted version of the original scale [45], which assesses young people’s decision-making skills.

Mental Health and Well-being

- The Depression Anxiety Stress Scale (DASS-21) [46] is a 21-item self-report scale designed to measure levels of symptoms of poor mental health in relation to three subscales (depression, anxiety, and stress).
- The Warwick Edinburgh Mental Well-being Scale (WEMWBS) [47] is a 14-item scale used to assess the mental well-being of respondents.

Academic Outcomes

- The Attitudes towards School scale [48] measures students’ attitudes and feelings towards their school environment (e.g., teachers, homework, grades, and learning).

An effort was also made to measure students’ academic performance through both self-reported and teacher-reported grades; however, these data did not correlate, giving rise to concerns about their validity. Due to the absence of standardized test scores, the data provided by both students and teachers were deemed insufficient and were, therefore, not included in the analysis

#### 2.4.2. Implementation 

Implementation was measured using indicators, which were taken from two primary measures: (i) Teacher Weekly Reports and (ii) a Student Review Questionnaire. Classroom observations with a subsample of schools (*n* = 6) were also undertaken and these were used to validate the self-reported measures by comparing the indicator scores across the relevant dimensions. The Teacher Weekly Reports were completed online by teachers each week following the delivery of each session. These questionnaires were designed to assess the implementation of each session from the teachers’ perspective (e.g., adherence to program content, suitability of the content for students, students’ engagement with the session, and an overall rating of the session). The Student Review Questionnaire was completed by intervention students at post-intervention. This questionnaire was designed to examine the implementation of the program from the students’ perspectives (e.g., attendance of specific sessions; their teacher’s Quality of Delivery of the program; their own response to the program; their overall rating of the program). Using these two implementation measures, a number of indicators were selected based on their representativeness of one of the four dimensions of implementation quality (e.g., Dosage, Adherence, Quality of Delivery, and Participant Responsiveness). Each indicator was scored separately, and these scores were then averaged within each dimension and converted to a percentage to produce an overall dimensional score (e.g., Total Dosage score, etc.). A reliability analysis was also carried out on the four dimension scores (Dosage, Adherence, Quality of Delivery, and Participant Responsiveness) and a high internal consistency was found (α = 0.86). Correlations were also completed between the four total dimension scores (Table 1). The four total dimension scores (%) were then averaged to produce an overall Total Implementation Quality score.

Total Implementation Quality = (Total Dosage + Total Adherence + Total Quality of Delivery + Total Participant Responsiveness)/4.(1)

This method of combining indicators across implementation dimensions to produce a total index score is based on similar methods used in previous studies [30,49,50]. To determine whether a school was high-implementing or low-implementing, the visual binning procedure in SPSS was performed by applying cutoffs at the mean and ±1 standard deviation level, which resulted in two identifiable groups (high/low). Dix and colleagues [30] carried out a similar statistical procedure when determining the implementation quality of the KidsMatter program in Australia. This process was completed with schools’ Total Implementation Quality score as well as for each of the four individual total dimension scores. Additional information on the selected indicators, the scoring process, and the visual binning procedure can be found elsewhere [35]. Descriptive statistics are used in Table 1 to show differences between schools in the high- and low-implementation groups across the four dimensions and for Total Implementation Quality.

### 2.5. Statistical Analysis 

All statistical analyses were completed using SPSS Statistical Software Package, IBM (version 26). Prior to analysis, the implementation data were linked to the student outcome data by school. Due to the clustered nature of the data, linear mixed models (LMM) were used to determine the linear relationships between the implementation data and student outcome data longitudinally. The combined Total Implementation Quality score was first used to determine how the implementation affected outcomes over time using a repeated measures LMM. In order to carry out this analysis, the dataset required restructuring into a long format prior to this analysis [51]. Given that there were identifiable differences between the scores at the baseline [34], the repeated-measures LMM needed to control for these differences [51]. The LMM included “School” and “Student ID” as the random effects, while “Time” was inputted as the repeated effect.

While the repeated-measures LMM is useful in providing information on whether or not there was a change over time, this type of analysis does not explicitly detect when this change occurs. Therefore, following this initial analysis, a more in-depth analysis was carried out to examine the differences between the three groups at the two time points: (i) post-intervention and (ii) 12-month follow-up separately, controlling for the pre-test scores.

For the LMM’s at post-intervention and 12-month follow-up, “Treatment Group” (high, low, control), based on the Total Implementation Quality score, was modeled as a fixed effect, while “School” was modeled as the random effect. “Gender” and “Baseline Scores” were modeled as covariates. The dependent variables included all student outcomes: social emotional skills, mental health, well-being, and academic performance at post-intervention and/or 12-month follow-up. The Bonferroni correction was applied to adjust for multiple comparisons and the a priori alpha level set for this study was 0.05.

In order to assess the relationships between the implementation dimensions and outcomes, LMMs were also carried out for each of the four individual dimensions. In these models, everything remained the same except for the fixed effect, which was replaced with the “Treatment Group” variable reflective of each dimension. For example, when assessing the relationship between dosage and the outcome variables, “Dosage Treatment Group” (high, low, control) was modeled as the fixed effect. These models were only run for those outcomes that were shown to be significant in the initial LMM analysis. The findings from this study are reported in compliance with the CONSORT 2010 statement for cluster randomized trials.

## 3. Results

### 3.1. Participants

Within this study, the analysis was carried out with three groups: (i) high-implementation, (ii) low-implementation, and (iii) control. Based on Total Implementation Quality, there were eight schools assigned to the high-implementation group (*n* = 169) and eight schools allocated to the low-implementation group (*n* = 143). The mean outcome scores for each group (high, low, and control) at each time point (pre-, post-, 12-month follow-up), based on Total Implementation Quality, are presented in Table 2. There were also 15 schools assigned to the control group (*n* = 345). Reported number of students (n) are based on baseline measurements. For numbers at post-intervention and 12-month follow-up, see Table 2.

### 3.2. Post-Intervention 

Mixed models were run on the relationship between the level of implementation quality and outcomes at post-intervention and the results can be found in Table 3. The findings of the linear mixed model for each of outcome variables at post-intervention, comparing the high-implementation group with both the low-implementation and control group, as well as the low-implementation group with the control group, are now presented.

#### 3.2.1. Social Emotional Skills 

Compared to control schools, high levels of implementation quality (but not low levels) were associated with significantly lower levels of avoidance coping (*β* = −1.53, 95% CI −2.58 to −0.48; *p* = 0.006), reduced expressive suppression (*β =* −0.95, 95% CI −1.88 to −0.01; *p* = 0.049), and significantly higher levels of social support coping (*β* = 1.20, 95% CI 0.031 to 2.09; *p* = 0.009) at post-intervention. Levels of implementation quality were not significantly associated with any other SEL outcome (all *p* > 0.05) when comparing the high-implementation group to control group. No significant differences were found between the low-implementation and control group for any of the social emotional skill outcomes (all *p* > 0.05).

#### 3.2.2. Mental Health and Well-being

Higher levels of implementation (but not low) were significantly associated with lower levels of stress (*β* = −2.1, 95% CI −3.73 to −0.47; *p* = 0.012) and depression (*β* = −2.0, 95% CI −3.73 to −0.03; *p* = 0.025). Levels of implementation quality were not associated with anxiety or well-being outcomes (both *p* > 0.05). No significant differences were found between the low-implementation and control groups for any of the mental health and well-being outcomes (all *p* > 0.05).

#### 3.2.3. Academic Outcomes

A significant difference was found between the high-implementation group compared to the low-implementation group for Attitudes towards School, with the high-implementation group demonstrating more positive attitudes towards school (*β* = 3.45, 95% CI 0.55 to 6.35; *p* = 0.022). No intervention effects were demonstrated for students’ attitudes toward school for the high- or low-implementation groups when compared to the control group (*p* > 0.05), though the difference between the high-implementation and control groups approached significance (*p* = 0.053).

### 3.3. Twelve-Month Follow-up 

Mixed models were again run for all of the variables, comparing the high-implementation group with both the low-implementation and control group and the low-implementation group with the control group. The results of the linear mixed model for key outcome variables at 12-month follow-up are shown in Table 4. Compared to control schools, high-implementation schools demonstrated significantly lower avoidance coping at 12-month follow-up (1.91 decrease, 95% CI −3.65 to 0.162; *p* = 0.033). No significant differences were found between the three groups for any of the other outcomes (all *p* > 0.05).

### 3.4. Implementation Dimensions 

Mixed models were run to compare the three groups (high, low, control) according to dimension group level across the variables that demonstrated significance during the initial mixed model analysis. Therefore, mixed models were run for: (i) Avoidance coping, (ii) Social Support coping, (iii) Expressive Suppression, (iv) Stress, (v) Depression, (vi) Attitudes towards School at post-intervention, and (vii) Avoidance coping at 12-month follow-up. Results of these LMMs according to dimensions at post-intervention and 12-month follow-up are reported in Table 5.

#### 3.4.1. Dosage

For Dosage at post-intervention, there were 10 schools in the high-implementation group and six in the low-implementation group. Dosage was significantly associated with two of the six intervention outcomes. Compared to the control group, a high dosage was associated with significantly lower levels of students’ Avoidance coping (1.32 decrease, 95% CI −2.30 to −0.345; *p* = 0.010) and Stress (2.0 decrease, 95% CI −3.52 to −0.470; *p* = 0.010). The dosage level for high-implementation schools was also related to decreased levels of avoidance at 12-month follow-up (2.15 decrease, 95% CI −3.75 to −0.555; *p* = 0.010). Levels of dosage were not associated with any other intervention outcomes (all *p* > 0.05).

#### 3.4.2. Adherence 

For Adherence, there were nine schools in the high-implementation group and seven in the low-implementation group. Levels of Adherence were significantly associated with two of the six intervention outcomes. Compared to the control group, high levels of Adherence were associated with lower levels of Avoidance coping (1.44 decrease, 95% CI −2.38 to −0.491; *p* = 0.005) and lower levels of Depression (1.60, 95% CI −3.19 to −0.004; *p* = 0.049). Levels of Adherence were not associated with any other intervention outcome (all *p* > 0.05).

#### 3.4.3. Quality of Delivery 

For Quality of Delivery, there were seven schools in the high-implementation group and nine in the low-implementation group. Levels of Quality of Delivery were significantly associated with all six intervention outcomes at post-intervention. Compared to the control group, high levels of Quality of Delivery were associated with lower levels of; avoidance coping (1.63 decrease, 95% CI −2.7 to 0.56; *p* = 0.005), suppressing emotions (1.0 decrease, 95% CI −1.97 to −0.041; *p* = 0.041), depression (2.15 decrease, 95% CI −3.93 to −0.36; *p* = 0.018), and stress (2.3 decrease, 95% CI −3.9 to −0.63; *p* = 0.007), and were associated with higher levels of social support coping (1.3 increase 95% CI 0.438 to 2.26; *p* = 0.004) and attitudes towards school (3.1 increase, 95% CI 0.65 to 5.54; *p* = 0.015). Compared to the low-implementation group, the high level Quality of Delivery group also demonstrated improved social support coping (1.28 increase, 95% CI 0.21 to 2.35; *p* = 0.019) and attitudes towards school (4.32 increase, 95% CI 1.55 to 7.08; *p* = 0.004). Quality of Delivery levels were also related to decreased levels of avoidance at 12-month follow-up for high-implementation schools (1.93 decrease, 95% CI −3.32 to −0.529; *p* = 0.009).

#### 3.4.4. Participant Responsiveness 

For Participant Responsiveness, there were eight schools in the high-implementation group and eight in the low-implementation group. Higher levels of Participant Responsiveness were associated with four of the six outcomes when compared to the control group. Schools with higher levels of Participant Responsiveness demonstrated decreases in avoidance (1.51 decrease, 95% CI −2.57 to −0.450; *p* = 0.007), suppressing emotions (1.03 decrease, 95% CI −1.97 to −0.091; *p* = 0.033) and stress (1.94 decrease, 95% CI −3.60 to −0.279; *p* = 0.022) as well as increased social support coping (1.17 increase, 95% CI 0.261 to 2.08; *p* = 0.012). Participant Responsiveness levels were also related to decreased levels of avoidance at 12-month follow-up for high-implementation schools (1.97 decrease, 95% CI −3.73 to −0.205; *p* = 0.030).

## 4. Discussion

The core aim of this study was to determine whether the level of implementation quality based on the Total Implementation Quality score of schools had a significant impact on program outcomes for students at post-intervention and/or 12-month follow-up. In the original MindOut c-RCT study, intervention students were found to have demonstrated significant improvements in a number of social emotional skills and mental health outcomes between pre- and post-intervention [34]. However, this outcome study did not take into account the varying levels of implementation quality of intervention schools, which is essential for understanding the program’s effectiveness [9]. Taking implementation quality into consideration, and assigning intervention schools to two separate groups (high- and low-implementation) dependent on their Total Implementation Quality score, the findings show that all outcomes that were found to be significant in the original study (social support coping, avoidance coping, suppressing emotions, depression, and stress) were only significant for those schools in the high-implementation group at post-intervention. Therefore, while the MindOut program was effective in producing positive outcomes for participants, this was only the case in schools that delivered the program to a high standard. Moreover, while the original study did not detect any significance between the control and intervention groups for Attitudes towards School, this study found that the high-implementation group scored significantly higher than both the low-implementation and control group for this outcome. Thus, after considering implementation, we can conclude that when MindOut is implemented as intended, the program can be successful in producing positive outcomes for participants. However, when the MindOut program is not implemented with high quality, the intended effects of the program are lost. These findings are in line with other studies on program implementation [9,10].

In order to understand the true effectiveness of a program, the quality of implementation needs to be considered [20,21,52,53]. If a majority of schools had implemented MindOut with poor quality, resulting in few or no positive outcomes, and the implementation quality had not been monitored, it would have been concluded that the program was ineffective, when in fact the lack of positive outcomes would likely be a result of poor implementation. By monitoring implementation, the risk of misinterpreting results like this is reduced and a better understanding of the conditions under which a program succeeds and/or fail can also be gleaned so that efforts can be made to maximize the quality of implementation and outcomes in the future [9,15,16].

Not only do the findings from this study demonstrate the importance of measuring implementation; they also highlight the importance of supporting the high-quality implementation of programs. Though a program may be theoretically sound, this does not ensure positive outcomes if the program is not implemented to a high standard [9,53]. Spending money, time, energy, and resources on programs that are not being implemented to a high standard is wasteful and disadvantageous for students, teachers and the education system [9]. Therefore, it is important that stakeholders commit to carrying out and supporting high-quality implementation of programs to ensure that these investments are worthwhile [5,9].

A further finding from this study was that only one of the outcomes measured (e.g., avoidance coping) was sustained at 12-month follow-up for the high-implementation group. While the MindOut program had an immediate impact on students’ social and emotional skills and mental health outcomes, the program was not able to produce long-term outcomes. Possible reasons for this drop-off effect will now be considered within the context of our study. Firstly, it is possible that the outcomes were not sustained at 12-month follow-up due to the timing of follow-up data collection, which took place during the spring semester of the year following implementation. Many students participating in the MindOut study would have been sitting their final-year ‘Leaving Certificate’ exams a few months after the time of the follow-up data collection. The pressures of education and the Leaving Certificate exams in Ireland are known to put added stress on adolescents and cause burnout [54,55]. Given that the majority of high-implementation school students (75%), in comparison to low-implementation schools (37%) and control schools (36%), would have been in the Leaving Certificate year at the time of the 12-month follow-up, it is possible that these students’ reported outcomes could have been negatively affected by this pressure. Secondly, given that MindOut is primarily a curriculum-based program, it is possible that its lack of integration at a whole-school level could have impacted its ability to sustain longer-term outcomes. The literature on the most effective strategies for school-based mental health promotion suggests that embedding SEL strategies into the daily practices of schools, across years and at a whole-school level, is likely to produce the best and most sustained outcomes for participants [2,13,32,56,57,58]. In order to enhance the probability of social and emotional skills being sustained in the long-term, they need to be intentionally taught, practiced, and reinforced on an ongoing basis [32,59,60,61,62]. By integrating strategies at a whole-school level (e.g., (i) curriculum; (ii) ethos and environment; and (iii) family and community) more opportunities can be created for students to learn and acquire these skills, increasing their likelihood of developing and sustaining positive outcomes.

An additional aspect of this study was to investigate whether or not individual dimensions of implementation quality influenced outcomes differently. Findings demonstrated that of the four dimensions assessed, Quality of Delivery had a significant impact on all of the six outcomes tested. Participant Responsiveness was the second most influential dimension, significantly impacting four out of six of the outcomes. Both Adherence and Dosage levels had a significant impact on two of the six dimensions at post-intervention. These findings show that, while all four dimensions studied play some role in the achievement of positive outcomes for participants, Quality of Delivery followed by Participant Responsiveness were the most influential dimensions. Given that a majority of previous studies assessing the impact of implementation quality on outcomes have used Adherence and/or Dosage as the primary implementation reference measurements, this finding is quite interesting [10,27,28]. Essentially, this finding reinforces previous studies that have examined implementation quality across multiple dimensions and have found that Quality of Delivery and Participant Responsiveness are just as important as Dosage and Adherence [14,22,26,29]. This indicates that, while Dosage and Adherence are essential parts of implementation quality and need to be upheld, the other dimensions should be given just as much attention. If this study had not assessed implementation quality using a composite score of all four dimensions, and instead used measures of Dosage or Adherence only, the findings would have told a different story. Likewise, if teachers deliver an entire program but fail in terms of Quality of Delivery (e.g., not prepared, engaging, enthusiastic, etc.), then it is likely that positive outcomes will not be achieved for students.

Given that Quality of Delivery and Participant Responsiveness have a strong influence on program outcomes, a greater focus should be placed on these aspects of implementation by strengthening the environment in which SEL programs are taught. The school climate has been identified as an important factor in the successful implementation of programs [63,64]. For example, positive student-teacher relationships have been shown to enhance their motivation, involvement, and participation in class [65,66,67], increasing students’ exposure to the program and the positive outcomes it is associated with. In this study, high-implementation schools demonstrated more positive attitudes towards school compared to both the low-implementation and control groups. It is possible that high-implementation students were exposed to a better school climate, which led to the development of stronger outcomes. Therefore, in introducing SEL programs into schools, teacher training and school implementation support should not only focus on the program itself, but also on the strategies needed for improving the school climate.

### 4.1. Implications for Research and Practice

The findings from this study can be used to inform the fields of research, practice, and policy. In terms of research, the study highlights the need for implementation measurement to be a key component of evaluation studies alongside the measurement of outcomes. It is essential that researchers take into consideration not only what is implemented but how it is implemented in order to fully understand what leads to a program’s effectiveness. It is also recommended that, when assessing implementation quality, researchers include measures of multiple dimensions to better understand their individual role in relation to outcomes and to get a more accurate and representative assessment of overall levels of implementation. In line with the recommendations of Dane and Schneider [15] and others [68], when measuring the implementation quality, multiple methods and multiple informants should be used and, where feasible, observational data should be included [26,69].

The study also indicates the need for researchers to engage in evaluation studies that can assess long-term program outcomes in addition to immediate outcomes. A majority of SEL program evaluations do not include long-term follow-up data and, therefore, it is difficult to determine whether the MindOut program is comparable to other SEL programs in terms of the long-term sustainability of outcomes.

In terms of practice and policy, these findings signify the importance of implementing programs to a high standard and ensuring that suitable strategies, resources, and policies are in place to support quality implementation. Failing to carry out and support high-quality implementation will likely diminish outcomes for participants, resulting in wasted time, money, and resources for all involved [5]. The study also suggests that curriculum-based programs may not be sufficient when determining how to achieve the best and most sustainable outcomes. Practitioners and key stakeholders are encouraged to consider embedding SEL practices into a whole-school approach, with curriculum-based programs being a key feature of this strategy. Embedding SEL strategies into the ethos and environment of the school, as well as linking to students’ lives outside of school (e.g., family and community), provides extended opportunities for students to practice and develop these skills, which will in turn create better outcomes that are longer-lasting. Given that Quality of Delivery and Participant Responsiveness were the most influential dimensions on outcomes, teacher training and support should focus on strategies for strengthening these aspects.

### 4.2. Strengths and Limitations 

The current study has several notable strengths that contribute to the value of the findings. This study employed a rigorous design, incorporating both outcome and process data for students in disadvantaged schools and carrying out a 3 × 3 (time × group) c-RCT. This study also included data on participants at 12 months post-intervention. By collecting long-term data, this study was able to determine the sustainability of outcomes for participants, which many other studies fail to do. A further strength of this study was the comprehensive measurement of implementation quality, which included multiple dimensions. A number of previous studies that have assessed implementation quality have limited their assessment to one or two dimensions. This study employed measures for four dimensions of implementation, allowing for a more inclusive and accurate interpretation of implementation quality. While there are several strengths in the research design of this study, it also has several limitations, which should be considered. The implementation indicators used in this study did not have established psychometric properties, but instead were based on implementation data that were collected from multiple sources (teachers and students). Furthermore, it was not possible to assess all five aspects of implementation quality as there were no suitable indicators for program differentiation. If given the chance to repeat this study, it would be preferable if measures of implementation were selected based on good psychometric properties and across all five dimensions of implementation quality [15]. Another limitation to this study is that all the data (outcome and process) were collected through self-reported measures, which poses a risk of participant response bias. It is recommended that implementation is captured through observational data over multiple occasions to increase reliability and reduce issues with response bias [69]. Although observational data were collected from schools in order to assess implementation quality, due to lack of time and resources, it was only possible to collect these data from a subsample of the schools (*n* = 6). Therefore, while these data could not be used within the main evaluation of implementation quality, they were used to validate the self-reported and teacher-reported data across the dimensions. Additional details on this process can be found in a previous study [35].

A final limitation of this study was the measure of academic outcomes, which was limited to the Attitudes towards School Scale. As reported under *Measures* in this paper, efforts were made to assess students’ grades through self-reported measures; however, due to concerns regarding their validity, and the lack of access to standardized testing scores within Irish schools, no appropriate measures of grades were available.

## 5. Conclusions

The current study contributes to the growing literature on the relationship between implementation quality and outcomes for school-based SEL programs. While well-developed, evidence-based programs are vital to the success of SEL initiatives, without the support of high-quality implementation it is unlikely that these programs will produce the promising outcomes expected [9,10,11]. The findings from this study support previous research demonstrating that positive program effects for participants are achieved only when there is high-quality implementation. Additionally, the study findings highlight the importance of measuring implementation quality across multiple dimensions of implementation and suggest that the dimensions of Quality of Delivery and Participant Responsiveness are equally important, if not more important, for achieving outcomes in school-based SEL programs. These findings add to the growing body of implementation research as they demonstrate the importance of the relationship between implementation quality and program outcomes and how this can be measured, while also contributing to the evidence base and an improved understanding of this relationship. These findings also have implications for future program delivery, highlighting the importance of ensuring strategies, resources, and policies are in place that support high-quality implementation in order for positive outcomes to be achieved.

## Figures and Tables

**Table 1:** Descriptive statistics (*n*, Means, and SDs) on implementation dimensions, by high- and low-implementation groups.

	High-Implementing Schools			Low-Implementing Schools		
Implementation Dimensions	*n*	*Mean (SD)*	*Range*	*n*	*Mean (SD)*	*Range*
Total Dosage	10	93.3% (2.9)	89.1–97.7%	6	74.2% (15.9)	45.8–85%
Total Adherence	9	87.3% (8.3)	79.3–100%	7	64.5% (11.0)	43.7–77.2%
Total Quality of Delivery	7	86.3% (4.0)	80.0–92.5%	9	67.9% (8.2)	56.5–79.5%
Total Participant Responsiveness	8	81.4% (4.6)	76–88.5%	8	68.7% (4.2)	62.3–74.5%
Total Implementation Quality	8	85.6% (3.9)	81.0–92.0%	8	71.6 (8.3)	52.7–78.4%

Notes: Mean = Average of combined indicators converted to a percentage; Correlations: Dosage/Adherence r = 0.78, *p* = 0.000; Dosage/Quality of Delivery r = 0.46, *p* = 0.073; Dosage/Participant Responsiveness r = 0.59, *p* = 0.017; Adherence/Quality of Delivery r = 0.21, *p* = 0.43; Adherence/Participant Responsiveness r = 0.62, *p* = 0.011; Quality of Delivery/Participant Responsiveness r = 0.55, *p* = 0.026.

**Table 2:** Mean outcome scores for high-implementation, low-implementation, and control groups at pre-, post-, and 12-month follow-up.

		High-Implementation M (SD)			Low-Implementation M (SD)			Control M (SD)		
		Pre- *N* = 169	Post- *n* = 125	Follow-up *n* = 116	Pre- *N* = 149	Post- *N* = 106	Follow-up *n* = 77	Pre *N* = 345	Post *N* = 251	Follow-up *n* = 220
**RSES**	Self-esteem	28.60 (5.2)	29.19 (5.3)	27.56 (5.5)	29.24 (5.0)	29.25 (5.2)	28.62 (5.0)	27.20 (5.5)	27.49 (5.4)	26.96 (5.2)
**TMMS**	Total Emotional Intelligence	69.15 (10.0)	81.78 (11.7)	77.88 (10.8)	69.41 (9.6)	81.96 (10.5)	79.93 (9.4)	67.83 (10.1)	79.27 (11.6)	79.04 (10.2)
	Subscale: Attention to Feelings	26.4 (4.8)	26.9 (5.0)	26.0 (5.0)	26.2 (4.8)	26.7 (3.9)	26.0 (4.1)	26.2 (4.6)	25.9 (4.8)	26.4 (4.9)
	Subscale: Emotional Clarity	25.7 (4.7)	26.1 (5.9)	24.6 (5.1)	25.9 (4.9)	26.7 (5.0)	25.9 (4.4)	24.7 (5.4)	25.2 (5.5)	25.1 (5.1)
	Subscale: Emotional Repair	28.0 (6.0)	29.1 (5.1)	27.0 (5.1)	28.2 (5.6)	28.6 (5.2)	28.3 (5.2)	27.9 (5.7)	28.2 (5.7)	27.7 (5.0)
**CSI**	Subscale: Avoidance	16.7 (6.0)	15.8 (4.9)	16.6 (5.5)	16.5 (5.8)	16.6 (5.5)	17.0 (6.1)	18.3 (6.2)	18.4 (5.7)	19.2 (5.7)
	Subscale: Problem-Solving	16.2 (5.2)	16.5 (5.1)	15.4 (4.7)	16.1 (5.0)	15.7 (5.0)	15.7 (4.7)	16.1 (5.3)	16.0 (5.0)	15.7 (5.1)
	Subscale: Social Support	12.05 (5.0)	13.7 (5.2)	13.4 (4.4)	12.6 (5.5)	12.7 (5.4)	13.3 (4.5)	13.4 (5.7)	13.1 (5.2)	13.4 (5.2)
**SEC-Q**	Social Self-efficacy	26.8 (6.2)	27.5 (6.6)	6.2 (6.0)	27.7 (6.1)	27.4 (6.1)	27.2 (6.2)	27.0 (6.0)	27.0 (6.3)	27.0 (6.3)
**ERQ**	Subscale: Reappraisal	26.4 (7.4)	27.0 (6.6)	25.5 (6.4)	26.1 (6.6)	25.9 (6.7)	25.5 (6.1)	25.9 (8.1)	25.8 (7.5)	25.0 (7.3)
	Subscale: Expressive Suppression	15.5 (5.3)	14.2 (4.2)	15.5 (4.2)	15.5 (5.2)	15.0 (4.3)	15.5 (4.7)	15.9 (5.3)	15.6 (4.8)	15.6 (4.6)
**AICQ**	Subscale: Asserting Influence	22.8 (5.9)	23.5 (6.0)	22.7 (6.1)	23.7 (5.7)	23.6 (5.4)	23.7 (5.2)	23.5 (5.8)	23.1 (5.7)	23.0 (6.3)
	Subscale: Conflict Resolution	21.4 (5.7)	22.1 (5.6)	21.4 (5.6)	22.2 (5.4)	22.3 (5.2)	22.1 (4.7)	21.9 (5.6)	22.1 (5.5)	22.3 (5.6)
**DMS**	Decision-Making	13.9 (3.6)	14.0 (3.3)	13.5 (3.7)	13.6 (3.1)	13.3 (3.3)	13.1 (3.2)	13.9 (3.3)	13.7 (3.4)	13.6 (3.4)
**DASS-21**	Stress	13.7 (8.8)	12.2 (8.3)	14.0 (7.8)	12.9 (9.4)	13.0 (8.9)	14.0 (8.0)	15.8 (9.8)	15.8 (9.6)	16.6 (9.5)
	Anxiety	12.04 (9.1)	9.9 (8.9)	12.3 (7.9)	11.2 (9.8)	10.8 (8.9)	12.0 (7.6)	13.8 (10.3)	13.1 (10.1)	14.0 (10.1)
	Depression	10.4 (9.6)	8.8 (9.1)	10.5 (7.3)	10.3 (9.7)	10.3 (8.9)	10.6 (7.5)	13.7 (11.0)	13.0 (10.2)	13.5 (9.1)
**WEMWBS**	Well-being	48.2 (9.9)	50.0 (9.4)	48.1 (9.1)	48.6 (11.1)	48.6 (10.7)	49.8 (9.8)	45.6 (12.3)	47.7 (11.0)	44.9 (10.5)
**ATS**	Attitudes toward School	57.7 (9.8)	58.6 (10.1)	55.0 (10.1)	55.6 (9.9)	53.6 (10.3)	54.6 (8.6)	55.6 (10.5)	53.9 (11.1)	55.3 (9.5)

Notes: 1. Group allocation (e.g., high and low) based on Total Implementation Quality score; 2. M = mean; SD = standard deviation; 3. RSES: Rosenberg Self-esteem scale, TMMS = Trait meta-mood Scale, CSI = Coping Strategy Indicator, SEC-Q = Self-Efficacy Questionnaire for Children, ERQ = Emotional Regulation Questionnaire, AICQ = Adolescent Interpersonal Competence Questionnaire, DMS = Decision-Making Scale ATS: Attitudes towards School Scale, DASS-21: Depression, Anxiety and Stress Scale, WEMWBS: Warwick Edinburgh Mental Well-being Scale.

**Table 3:** Mixed model results of the relationship between the level of implementation quality and outcomes at post-intervention.

	Models with the Full Sample and High-Implementation Group as Reference				Models Without High-Implementation and With Low-Implementation Group as Reference	
	*Control*		*Low*		*Control*	
Dependent Variable	*t*	*p*	*t*	*p*	*t*	*p*
Self-esteem	−1.47	0.142	−0.480	0.632	−0.834	0.405
Total Emotional Intelligence	−1.09	0.286	−0.021	0.984	−1.186	0.249
Subscale: Attention to Feelings	−1.56	0.132	−0.238	0.814	−0.957	0.351
Subscale: Emotional Clarity	−0.338	0.738	0.891	0.380	−1.46	0.146
Subscale: Emotional Repair	−1.08	0.29	−0.566	0.576	−0.37	0.713
Subscale: Avoidance ^a^	3.03	0.006**	0.806	0.427	1.89	0.060
Subscale: Problem Solving	−0.477	0.638	−0.98	0.335	0.697	0.493
Subscale: Social Support	−0.264	0.009**	−1.92	0.056	−0.217	0.831
Social Self-efficacy	−0.56	0.58	−0.73	0.47	0.290	0.775
Subscale: Reappraisal	−1.76	0.078	−1.44	0.151	−0.058	0.954
Subscale: Expressive Suppression ^a^	2.06	0.049*	1.14	0.262	0.717	0.480
Subscale: Asserting Influence	−1.04	0.297	−0.181	0.856	−0.788	0.431
Subscale: Conflict Resolution	−0.254	0.799	−0.342	0.733	0.040	0.968
Decision-Making	0.298	0.770	−0.949	0.354	1.31	0.192
Stress ^a^	2.10	0.035*	1.06	0.864	1.07	0.707
Anxiety ^a^	1.63	0.120	0.678	0.505	0.778	0.449
Depression ^a^	2.25	0.025*	1.24	0.215	0.688	0.492
Well-being	−0.54	0.597	−1.40	0.177	1.05	0.310
Attitudes toward School	−2.04	0.053	−2.45	0.022*	0.826	0.425

Notes: * *p* < 0.05; ** *p* < 0.01; ^a^ Reversed scoring outcomes; Group allocation (e.g., high and low) based on Total Implementation Quality score.

**Table 4:** Mixed model results of the relationship between the level of implementation quality and outcomes at 12-month follow-up.

	Models With the Full Sample and High-Implementation Group as Reference				Models Without High-Implementation and With Low-Implementation Group as Reference	
	*Control*		*Low*		*Control*	
Dependent Variable	*t*	*p	*t*	*p*	*t*	**p
Self-esteem	−0.380	0.707	0.709	0.484	−1.53	0.145
Total Emotional Intelligence	0.967	0.342	1.78	0.084	−0.552	0.581
Subscale: Attention to Feelings	0.357	0.724	0.022	0.982	0.211	0.835
Subscale: Emotional Clarity	0.964	0.344	1.40	0.171	−0.862	0.389
Subscale: Emotional Repair	1.32	0.186	1.80	0.072	−0.770	0.442
Subscale: Avoidance ^a^	2.24	0.033	0.251	0.803	1.82	0.081
Subscale: Problem Solving	0.538	0.591	0.517	0.605	0.002	0.998
Subscale: Social Support	−0.967	0.342	−0.554	0.584	−0.300	0.767
Social Self-efficacy	1.32	0.187	0.756	0.450	0.380	0.704
Subscale: Reappraisal	−0.844	0.399	0.058	0.954	−0.822	0.412
Subscale: Expressive Suppression^a^	−0.120	0.905	0.071	0.943	−0.229	0.819
Subscale: Asserting Influence	−0.088	0.930	0.865	0.388	−1.00	0.318
Subscale: Conflict Resolution	0.840	0.410	0.104	0.918	0.634	0.534
Decision-Making	0.091	0.928	−0.754	0.458	1.04	0.298
Stress ^a^	1.73	0.098	0.551	0.586	0.867	0.396
Anxiety ^a^	0.874	0.390	0.257	0.799	0.457	0.652
Depression ^a^	1.82	0.082	0.357	0.724	1.40	0.177
Well-being	−1.46	0.157	0.648	0.523	−1.94	0.068
Attitudes toward School	0.732	0.471	0.011	0.991	0.631	0.535

Notes: * *p* < 0.05; ** *p* < 0.01; ^a^ Reversed scoring outcomes; Group allocation (e.g., high and low) based on Total Implementation Quality score.

**Table 5:** Mixed model results across dimensions with high-implementation group as reference at post-intervention and follow-up.

Post-Intervention		*Control*		*Low*	
Variables	Dimensions	*t*	*p	*t*	**p
CSI: Avoidance	Dosage	2.80	0.010*	0.094	0.926
	Adherence	3.15	0.005**	0.714	0.481
	Quality of Delivery	3.16	0.005**	1.08	0.288
	Participant Responsiveness	2.95	0.007**	0.702	0.489
CSI: Social Support	Dosage	−1.82	0.084	−0.477	0.638
	Adherence	−1.48	0.155	0.359	0.723
	Quality of Delivery	−2.91	0.004**	−2.35	0.019*
	Participant Responsiveness	−2.53	0.012*	−1.71	0.089
ERQ: Expressive Suppression	Dosage	1.58	0.127	0.053	0.958
	Adherence	1.51	0.142	−0.157	0.876
	Quality of Delivery	2.15	0.041*	1.23	0.228
	Participant Responsiveness	2.25	0.033*	1.40	0.171
DASS-21: Stress	Dosage	2.57	0.010*	1.09	0.277
	Adherence	1.70	0.090	−1.04	0.300
	Quality of Delivery	2.71	0.007**	1.40	0.163
	Participant Responsiveness	2.30	0.022*	0.668	0.505
DASS-21: Depression	Dosage	1.63	0.105	−0.108	0.914
	Adherence	1.97	0.049*	0.639	0.523
	Quality of Delivery	2.37	0.018*	1.45	0.148
	Participant Responsiveness	1.82	0.070	0.487	0.627
Attitudes towards School	Dosage	−0.490	0.628	−0.785	0.439
	Adherence	−0.146	0.885	−0.052	0.959
	Quality of Delivery	−2.63	0.015*	−3.22	0.004**
	Participant Responsiveness	−1.14	0.265	−1.75	0.092
**Follow-up**		***Control***		***Low***	
**Variables**	**Dimensions**	***t***	***p***	**t**	***p***
CSI: Avoidance	Dosage	2.76	0.010*	10.03	0.312
	Adherence	1.89	0.068	−10.00	0.324
	Quality of Delivery	2.88	0.009**	−10.2	0.242
	Participant Responsiveness	2.28	0.030*	0.371	0.714

Notes: * *p* < 0.05; ** *p* < 0.01; Mixed models only completed for outcomes found to be significant in original analysis; CSI = Coping Strategy Indicator, ERQ = Emotional Regulation Questionnaire, DASS-21: Depression, Anxiety, and Stress Scale.
